# Expert Guidelines on the Use of Cariprazine in Bipolar I Disorder: Consensus from Southeast Asia

**DOI:** 10.3390/healthcare13111304

**Published:** 2025-05-30

**Authors:** Ahmad Hatim Sulaiman, Mustafa M. Amin, Jin Kiat Ang, Roger Ho, Nik Ruzyanei Nik Jaafar, Chong Guan Ng, Adhi Wibowo Nurhidayat, Pongsatorn Paholpak, Pornjira Pariwatcharakul, Thitima Sanguanvichaikul, Eng Khean Ung, Natalia Dewi Wardani, Brian Yeo

**Affiliations:** 1 Department of Psychological Medicine, Faculty of Medicine, Universiti Malaya, Kuala Lumpur 50603, Malaysia; chong_guan@um.edu.my; 2 Department of Psychiatry, Faculty of Medicine, Universitas Sumatera Utara, Medan 20222, Indonesia; mustafa.mahmud@usu.ac.id; 3 Department of Psychiatry, Faculty of Medicine and Health Sciences, Universiti Putra Malaysia, Serdang 43400, Malaysia; jinkiat@upm.edu.my; 4 Department of Psychological Medicine, Yong Loo Lin School of Medicine, National University of Singapore, Singapore 117597, Singapore; pcmrhcm@nus.edu.sg; 5 Division of Life Science, Hong Kong University of Science and Technology, Clear Water Bay, Hong Kong, China; 6 Department of Psychiatry, Hospital Canselor Tuanku Muhriz, Faculty of Medicine, Universiti Kebangsaan Malaysia, Kuala Lumpur 56000, Malaysia; ruzyanei@hctm.ukm.edu.my; 7 Department of Psychiatry, Faculty of Medicine, Universitas Islam Negeri Syarif Hidayatullah Jakarta, South Tangerang 15412, Indonesia; adhi.wibowo@uinjkt.ac.id; 8 Department of Psychiatry, Khon Kaen University, Khon Kaen 40002, Thailand; ppaholpak@kku.ac.th; 9 Department of Psychiatry, Faculty of Medicine Siriraj Hospital, Mahidol University, Bangkok 10700, Thailand; pornjirap@gmail.com; 10 Department of Psychiatry, Somdet Chaopraya Institute of Psychiatry, Bangkok 10600, Thailand; thitima.sanguanvichaikul@gmail.com; 11 Adam Road Medical Centre, Singapore 269695, Singapore; drkenung@yahoo.com.sg; 12 Department of Psychiatry, Diponegoro University, Semarang 50275, Indonesia; anatdew@gmail.com; 13 Mount Elizabeth Medical Centre, Singapore 228510, Singapore; brianyeoclinic@yahoo.com.sg

**Keywords:** bipolar I disorder, bipolar depression, bipolar mania, mixed episode, cariprazine, Southeast Asia

## Abstract

**Background/Objectives**: Cariprazine, a D3/D2 partial agonist, is one of the few recommended treatment options for bipolar 1 disorder (BP1D) in Southeast Asia. This study aims to generate insights from leading experts on the safe and effective use of cariprazine for BP1D, specifically by formulating practical recommendations not thoroughly covered in the existing literature. **Methods**: A formal consensus methodology using the modified RAND/UCLA Appropriateness Method was employed to develop consensus recommendations. The methodology included a targeted literature search, creation of clinical scenarios, two rounds of rating of the appropriateness of each scenario on a nine-point Likert scale by an expert panel of psychiatrists from Southeast Asia (*n* = 13), and a face-to-face discussion among the expert panel between the two rounds of rating. In the absence of disagreement, scenarios were classified as appropriate (7–9), equivocal (4–6), or inappropriate (1–3) based on median scores. Clinical scenarios were subsequently converted to consensus recommendations upon approval by the expert panel. **Results**: Most experts recommended a 4–8-week trial of cariprazine for bipolar depression (85%) and 3–4 weeks for acute mania/mixed (71%). For longer treatment, 61.5% and 69% recommended >1 year for acute mania/mixed and bipolar depression, respectively. Cariprazine was also considered suitable as first-line therapy, including for first-episode bipolar depression (Mdn: 8, IQR: 7–9) and first-episode mania (Mdn: 8; IQR: 8–9). **Conclusions**: The consensus recommendations may serve as practical guidance for clinicians to make informed decisions regarding the management of adult patients with BP1D, while considering the preferences and circumstances of individual patients.

## 1. Introduction

Bipolar I disorder (BP1D) is a chronic mental health condition that presents predominantly with mania and differential depressive symptoms and episodes [1,2]. The global lifetime prevalence of about 1.0% is based on high-income countries, with variable estimates for middle to low-income countries [1,2]. In Southeast Asia, large-scale epidemiologic studies are still limited [3,4]. Epidemiologic data from Singapore show a lifetime weighted prevalence of 3.1% for bipolar spectrum disorder, 1.5% for bipolar I, 0.03% for bipolar II, and 1.6% for subthreshold bipolar disorder [4].

Assessment poses challenges. A working diagnosis requires a longitudinal assessment supplemented by corroboration from family. Diagnostic delay can happen especially in heterogenous cases without the classic presentation of mania, such as in mixed states [2,5,6]. Mixed states may predict poorer pharmacotherapeutic response, often warranting combination therapy or polypharmacy [7,8]. Prognosis tends to worsen with comorbidities such as substance use and anxiety disorders, which occur more in bipolar disorder than in unipolar depression. Presence of concomitant mental health conditions markedly increases the risk of suicide [9]. Chronicity and frequent relapse worsen the burden of illness, with prevalence of the latter at 25.6% reported in one large retrospective cohort study [2,10]. Progressive neurobiological changes (i.e., neurostructural, cognitive, neurochemical) are hypothesized to result from illness duration and number of previous episodes [1,11].

Consistent with the purported pathophysiology of bipolar disorder, standard medications act on different neurochemical systems, including serotonergic, dopamine, glutamatergic, and GABAergic, which influence downstream signal transduction sites beyond neurotransmitter receptors (i.e., phosphatidyl inositol system, G proteins, GSK-3, PKC), cascading to influence regulation of gene expression for growth factors and neuronal plasticity [12].

Cariprazine, a treatment for BP1D, acts as a D3/D2/5HT1A partial agonist and as a 5HT2A/α1/α2 antagonist [12]. It differs from other serotonin/dopamine antagonists/partial agonists with its potent action on D3 receptors, exceeding dopamine itself [12]. There is a differential effect: blockade at higher doses, stimulant at lower [12]. This D3 antagonism/partial agonism aids in managing mania and schizophrenia when postsynaptic blockade of D3 receptors in limbic regions reduces manic and psychotic symptoms [12]. Lower doses improve depression and anxiety through antagonist/partial agonist influence on the presynaptic D3 autoreceptors within the ventral tegmental area (VTA) [12]. Through disinhibition, dopamine reaches the postsynaptic D1 receptors in the prefrontal cortex, addressing the hypodopaminergic state implicated in low mood, energy, and motivation, alongside cognitive problems in depression and negative symptoms in schizophrenia [12].

The dynamic mechanism of cariprazine accounts for its therapeutic efficacy across the entire bipolar disorder spectrum, from acute bipolar mania to acute bipolar depression, including mixed states, with equipotency across both mania and depression mitigating development of contrapolar symptoms [12,13,14]. Clinical practice guidelines (CPGs) like Canadian Network for Mood and Anxiety Treatments (CANMAT)/International Society for Bipolar Disorders (ISBD) guidelines also highlight the role of cariprazine as a first-line treatment for acute mania and bipolar depression with care towards potential adverse effects, such as nausea, akathisia, restlessness, and extrapyramidal symptoms [14,15,16]. Potent cytochrome P450 3A4 (CYP3A4) inhibitors and inducers are contraindicated for use of cariprazine [15,16]. Other atypical antipsychotics recommended for the treatment of BP1D include lurasidone and lumateperone for bipolar depression, aripiprazole and risperidone for acute mania, and quetiapine for both poles of BP1D, among others [14]. Cariprazine exhibits a more favorable metabolic profile compared to quetiapine and risperidone and demonstrates comprehensive efficacy across both acute mania and bipolar depression, in contrast to aripiprazole, lurasidone, and lumateperone [15,16].

Beyond cariprazine’s inclusion in CPGs, literature on psychopharmacologic applications, particularly in the Southeast Asian region, is limited. This study aims to generate insights from key experts on the safe and effective use of cariprazine for BP1D, specifically by formulating practical recommendations not thoroughly covered in the existing literature, i.e., appropriate patient characteristics, dosing, and duration of therapy; role of concomitant medications; and tolerability.

## 2. Materials and Methods

The modified RAND Corporation/University of California Los Angeles (RAND/UCLA) Appropriateness Method (RAM) was employed for consensus development because it effectively integrates both scientific evidence and expert opinion [17]. This contrasts with other formal consensus methods, such as the Delphi method, nominal group technique, and consensus development conference, which primarily rely on expert opinion [17]. Furthermore, the modified RAM was also used previously in consensus development in the field of psychiatry [18,19,20].

The consensus development study design was formulated and facilitated by a chair and co-chair, who also oversaw the discussion and implementation processes. Additionally, the chair and co-chair managed the selection of psychiatrists (*n* = 13) from countries in Southeast Asia, specifically from Indonesia, Malaysia, Singapore, and Thailand. The selection criteria for the panel are based on subject matter expertise and experience in the management of BP1D using cariprazine.

**Figure 1:**
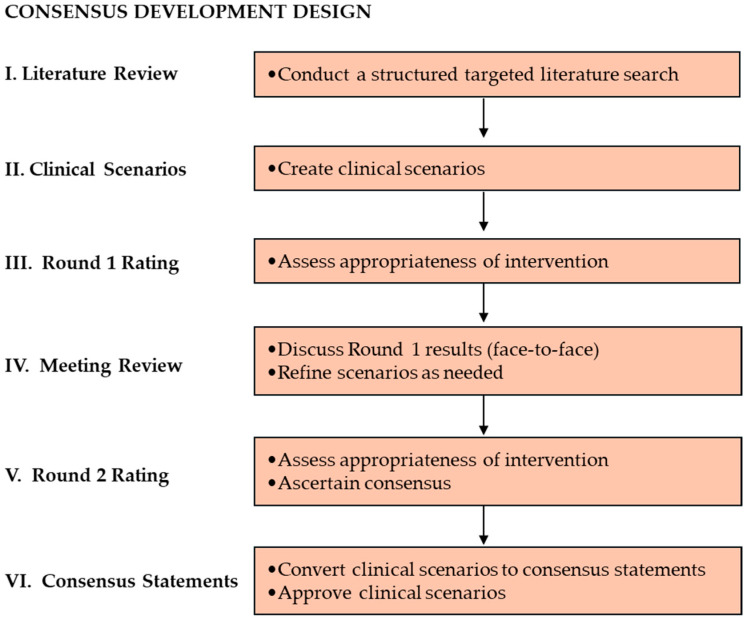
Overview of the Modified RAM study methodology.

Figure 1 illustrates the Modified RAM methodology, which commenced with a targeted literature review to identify key evidence. This was followed by the development of clinical scenarios that describe patient characteristics and clinical features. Each clinical scenario was evaluated for the appropriateness of treatment in two rounds. A face-to-face discussion was conducted between these two rounds of evaluation. After analysis of the results, the clinical scenarios were converted to consensus statements.

### 2.1. Targeted Literature Review

**Figure 2:**
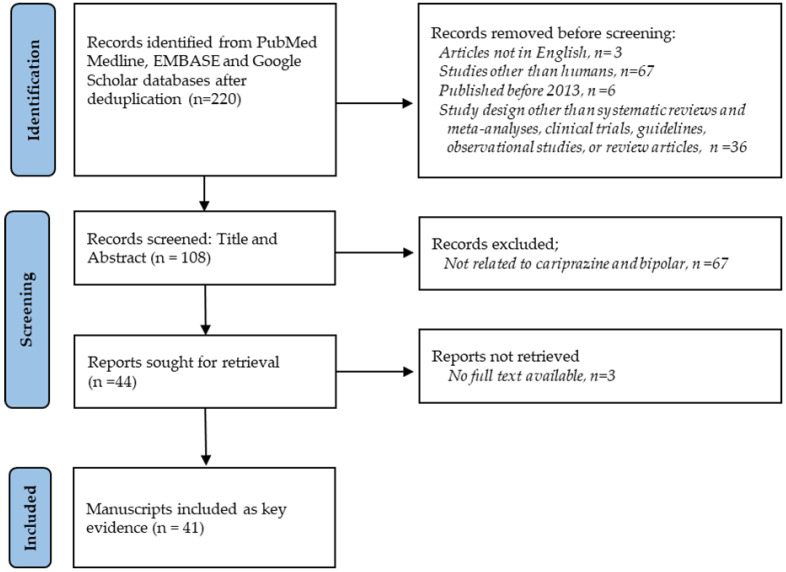
Literature search flow diagram.

In the process of consensus development, a structured targeted literature review (TLR) was conducted to identify manuscripts serving as key evidence for the creation of clinical scenarios [18,19]. These manuscripts were also shared to all panelists to facilitate rating of appropriateness of clinical scenarios and for discussion. The structured TLR method involved devising a systematic search strategy through several steps [21]. Initially, relevant keywords and terms were identified, including ‘cariprazine’, ‘bipolar disorder’, ‘mania’, and ‘bipolar depression’. Subsequently, a structured TLR search was executed across the following databases: PubMed Medline, EMBASE, and Google Scholar. Boolean and proximity operators (e.g., AND, OR, NOT) were utilized to combine search terms and refine search outcomes. For instance, the following search strings were employed in PubMed Medline: “cariprazine AND (“Bipolar Disorder” [Mesh] OR mani* OR depress*)”. Eligibility criteria were established to filter results, with studies deemed eligible if they were published in a peer-reviewed journal in English from 1 January 2013 to 30 June 2023. Systematic reviews, clinical trials, guidelines, observational studies, and review articles were included. The initial search identified a total of 220 articles, which were narrowed down to 108 after applying filters for study design, date, species, and language. Title and abstract screening were conducted on these 108 articles, resulting in the exclusion of 67 articles that were not related to cariprazine in bipolar disorder, while 3 articles were not retrieved due to the unavailability of full texts. Ultimately, 41 manuscripts were included as key evidence for consensus development. Figure 2 illustrates the flow of the studies from search to inclusion based on the PRISMA flow diagram [22].

### 2.2. Clinical Scenarios

Clinical scenarios, which include patient characteristics and clinical features that guide treatment decisions, were developed by the chair and co-chair based on the key evidence obtained. These scenarios were subsequently distributed to the expert panel for evaluation through two rounds of rating.

### 2.3. First Round of Rating

The first round of rating was completed by the expert panel via a private online survey form. The panelists were instructed to assess the appropriateness of the statement or intervention using a 9-point Likert scale (1 = strongly disagree; 9 = strongly agree). The rating was performed independently by each panelist. Additionally, panelists were encouraged to utilize the key evidence from the targeted literature search. The results of the first round of rating for each clinical scenario were summarized using the median and interquartile range.

### 2.4. Meeting Review

The expert meeting was convened in person over one day in October 2023 and was facilitated by the chair and co-chair. The results of the first round of rating were shared to all expert panel members. Participants discussed individual perspectives on the appropriateness of the intervention for each clinical scenario.

### 2.5. Second Round of Rating

In a manner consistent with the initial round, the second round of evaluations was conducted independently by participants through a private online survey form. The outcomes of this second round of evaluations for each clinical scenario were summarized using the median and interquartile range. Additionally, assessments of disagreement and the appropriateness of interventions were performed at this stage. A clinical scenario is deemed to exhibit ‘disagreement’ among raters when at least one-third of the panelists assign a rating within the bottom three points of the 9-point Likert scale (1 to 3), while simultaneously at least one-third of the panelists assign a rating within the top three points (6 to 9) [17,18,19]. In the absence of ‘disagreement’, a scenario with a median score of 7–9 was considered ‘appropriate’ (indicating that benefits outweigh risks); a median score of 4–6 was deemed ‘equivocal’ or ‘uncertain’; and a median score of 1–3 was considered ‘not appropriate’ (indicating that risks outweigh benefits) [17,18,19].

### 2.6. Consensus Recommendations

The results of the second round of evaluations regarding the appropriateness of treatment were subsequently transformed by the chair into consensus recommendations, which were reviewed and approved by all members of the expert panel.

## 3. Results

The results are presented into two main parts: (1) disease impact, problems and challenges with BP1D, and (2) recommendations for best practice. The recommendations are further subdivided into general recommendations, consensus statements for bipolar depression, and acute mania/mixed episodes, cariprazine dose recommendations, treatment duration, and lastly, management of treatment-emergent adverse events.

### 3.1. Bipolar 1 Disorder: Impact, Problems, and Challenges

#### 3.1.1. Clinicians’ Perception of the Disease’s Impact on Patients

**Figure 3:**
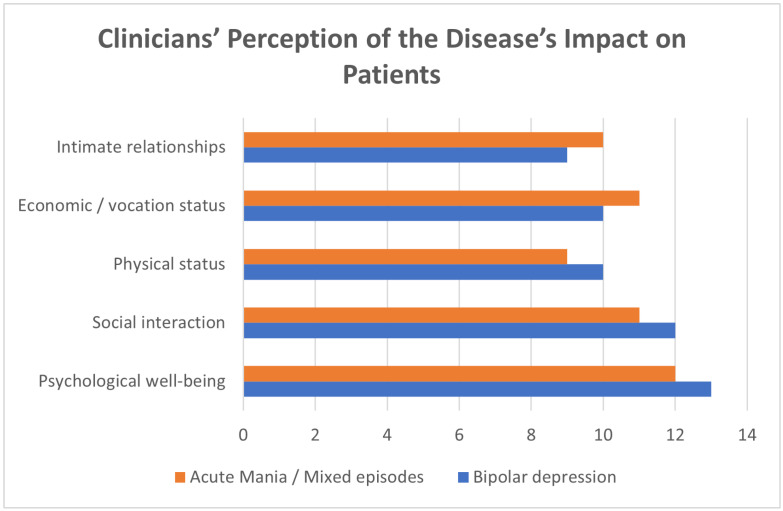
Clinician’s perception of the disease impact of both bipolar depression and acute mania/mixed episodes based on their clinical encounters.

Clinicians recognize how the disease affects psychological, social, physical, economic/vocational, and relational aspects of patients’ lives. Figure 3 illustrates clinician’s perception of the disease impact of both bipolar depression and acute mania/mixed episodes based on their clinical encounters.

#### 3.1.2. Challenges in Screening and Diagnosis of BP1D

Some of the challenges encountered in the screening, assessment, and diagnosis of BP1D include:

- Resources. The high patient load paired with inadequate human resource compounds the limited time of consultation. Added human resources that can mitigate time constraints in questionnaire administration are also limited.
- Psychiatric history. Incomplete history taking may lead to missed detection of subthreshold symptoms, manic episodes, and mixed features, as gathering of longitudinal data is necessary to identify the polarity index.
- Patient reliability. Patients may have an inclination to omit hypomanic states in their histories as they are often productive during these phases. Illness denial also contributes to delayed clinical consult.
- Rating scales. Screening tools, questionnaires and rating scales are often underutilized due to practicality issues. The possibility of rating scales not capturing the overall picture of a patient’s mood, such as the Montgomery–Åsberg Depression Rating Scale (MADRS) that may overlook bipolar 1 depression, risks misdiagnosis and outweighs any benefit to their use.
- Differential diagnoses. The differentiation of affective disorders with psychotic features from primary psychotic or thought disorders are also a notable diagnostic challenge. Challenges in assessment also include differentiation of bipolar disorder from unipolar depression (i.e., major depressive disorder with mixed features) and substance-induced mood disorder, as well as differentiation of bipolar 1 from bipolar 2 disorder.
- Comorbidities. The presence of personality disorders (e.g., borderline personality disorder) or a history of trauma with or without stress/trauma-related disorders can complicate assessment. The clinical presentation of bipolar disorder also tends to be atypical when substance use disorder is present.

#### 3.1.3. Challenges in Management of BP1D

Challenges in BP1D management involve patient, treatment, and health system factors.

- Treatment adherence. Poor insight into their condition and denial of their illness often lead to poor adherence or engagement, and sometimes outright refusal, to the treatment plan.
- Pharmacologic management. Prompt medication effectiveness in instances where rapid response is needed while preventing development of contrapolar symptoms is a prominent concern. Clinicians may have difficulty choosing appropriate medication for their patients. Polypharmacy tends to prevail in symptom management. Tolerability issues including treatment-related adverse effects also affect patients’ treatment adherence.
- Healthcare system resources. With a limited number of psychotherapists across countries, some rely solely on pharmacotherapy-based treatments if psychotherapy services are not available. Problems with access to affordable medications and safety monitoring measures, as well as a shortage of suitable inpatient facilities are also concerns in the region.

### 3.2. General Recommendations on Best Practices for Screening, Diagnosis and Management of BP1D

Raising public awareness on the importance of early consultation and symptom reporting can promote early screening, assessment, and management.

Evidence-based research on treatment options, peer discussions and reviews, and latest clinical practice guidelines is indispensable to clinician/specialist education. Awareness training on the identification mixed states of BP1D and determining the appropriate psychopharmacologic option suitable for patients are key areas for education and training.

In the clinical setting, tactfully eliciting a comprehensive history to identify and define mood episodes, including polarity, frequency and severity, and their related symptoms (i.e., aggression, high-risk behavior) in a non-stigmatizing way needs to be reinforced. Close monitoring and observation of mood episodes are also essential in assessment.

Balancing efficacy and safety/tolerability from acute to maintenance phase of treatment across depressive and manic episodes is a guiding principle in choosing the most suitable and appropriate medication for each individual. Management also includes provision of psychoeducation for patients and their families to maximize engagement and treatment adherence to the treatment.

To further address challenges, increasing human resources is necessary to respond to the high and growing demand for psychiatric services. Access to functional neuroimaging as an adjunct diagnostic tool may further enhance understanding and knowledge of the disorder.

### 3.3. Consensus Statements on Cariprazine’s Place in Therapy: Patient Characteristics to Consider

Table 1 and Table 2 summarize the final consensus statements in bipolar I depression and in acute mania/mixed episodes determined through the modified RAM.

### 3.4. Cariprazine Dose Recommendations

#### Bipolar Depression

**Figure 4:**
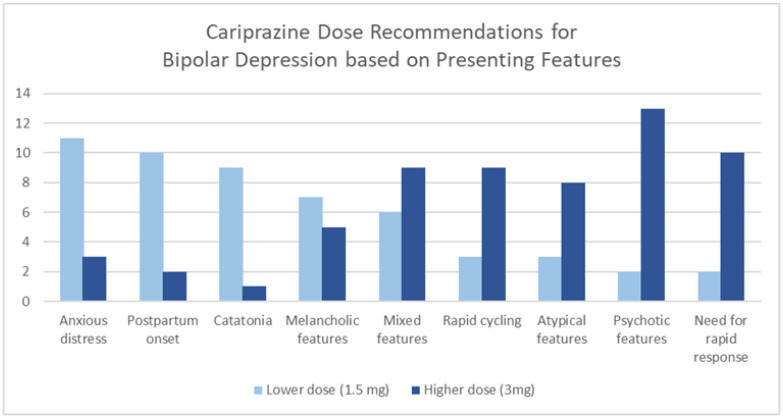
Cariprazine dose recommendations for bipolar depression based on presenting features.

Figure 4 illustrates cariprazine dose recommendations for bipolar depression based on the clinical features that direct treatment decisions.

Other indications/considerations in the dose of cariprazine in the treatment of bipolar depression are presented in Table 3.

### 3.5. Treatment Duration

#### 3.5.1. Bipolar Depression

Eighty-five percent (85%) of the expert panel (*n* = 11) recommended that the minimum duration of cariprazine treatment to consider an effective trial is 4–8 weeks, with the remainder (*n* = 2) suggesting less than 4 weeks.

Recommendations on the duration of cariprazine use varied, with 61.5% recommending treatment beyond 1 year or indefinitely.

#### 3.5.2. Acute Mania/Mixed Episodes

The minimum duration of cariprazine treatment to consider an effective trial in acute mania or mixed episodes was 3 to 4 weeks based on the recommendations of 61.5% (*n* = 8) of the expert panel, with 31% (*n* = 4) preferring 4 to 8 weeks and the remainder less than 3 weeks (*n* = 1).

For patients who have achieved remission, cariprazine can be considered for more than a year or indefinitely according to 69% of the expert panel. Thirty-one percent (31%) (*n* = 4) would continue cariprazine for less than or equal to a year.

For acute mania/mixed episodes, 71% of the expert panel recommended 3–4 weeks duration as the minimum duration of cariprazine treatment to consider an effective trial.

### 3.6. Treatment-Emergent Adverse Events (TEAEs): Practical Recommendations

Approach to management of TEAEs associated with cariprazine such as tardive syndromes and sleep disturbances are outlined in Table 4.

## 4. Discussion

Nuances in effective management of BP1D necessitates employing treatment that is both efficacious and safe, preferably achieving early response without inducing to a contrapolar state. To decrease the tendency for polypharmacy, it would be beneficial to have pharmacologic agents that can address the various ways in which BP1D presents clinically alongside its common comorbidities.

The expert panel deemed 26 clinical considerations in the clinical utility of cariprazine in the management of bipolar depression (Table 1) and 29 clinical considerations in the management of acute mania/mixed episodes (Table 2) appropriate.

The panel agreed that cariprazine is appropriate in most cases as monotherapy in bipolar depression, acute mania, and mixed episodes. In clinical trials, about half of patients presenting with mania significantly improved and responded with a single agent within 3 to 4 weeks [14,23]. However, while monotherapy and oral administration are preferred where the individual is agreeable, these may not be sufficient especially in cases of severe psychosis where additional medication is often necessary [13].

Cariprazine was recommended suitable for the treatment of clinical symptoms such as anhedonia, anxiety, catatonia, and rapid-cycling presentations. Management of BP1D with comorbidities like attention-deficit hyperactivity disorder (ADHD), impulse control disorder, and personality disorder were considered appropriate for use of cariprazine. Preclinical studies, based on cariprazine’s pharmacodynamic profile, indicate that it has the potential to improve both anhedonia symptoms and cognition [24]. The high D3 affinity of cariprazine may confer procognitive effects, addressing residual cognitive symptoms following resolution of a mood episode and possibly mitigating the rapid relapse and enduring chronicity of bipolar disorder [25,26,27]. A recent post hoc analysis illustrated statistically significant improvement in cognitive symptoms among patients with bipolar depression on MADRS and Reisberg Functional Assessment Screening Tool (FAST) cognitive subscale, as well as bipolar mania in Positive Scale, Negative Scale, and General Psychopathology Scale (PANSS) cognitive subscale versus placebo [28].

Efficacy studies on medications that specifically address anxiety symptoms in patients with bipolar disorder are limited, thus leaving a common but difficult and pressing issue unaddressed. A post hoc analysis of pooled data from two double-blind, randomized, placebo-controlled, multicenter, parallel-group, fixed-dose phase 3 studies in adults with BP1D and a major depressive episode found that cariprazine improved symptoms of anxiety and depression in patients with bipolar depression and higher baseline anxiety in MADRS total score, Hamilton Anxiety Rating Scale (HAM-A) total score and subscale scores [29]. Although the study limitations restrict from drawing definitive generalizations about effectiveness, the initial data show promise [29]. Naturalistic studies and randomized controlled trials show improvement of acute catatonic and psychotic symptoms with second-generation antipsychotics. Nevertheless, the risk of uncommon adverse effects, including antipsychotic-induced catatonic states, remains [30]. Management of rapid cycling bipolar disorders (RCBD) is supported by the D2/D3 partial antagonism of cariprazine, which may produce more robust effects compared to full antagonists on the high mood instability typical of RCBD. Data on its use in ADHD are still limited (i.e., case report) [31]. Several case reports and series have demonstrated the effectiveness of cariprazine in regulating impulsivity [32,33,34]. Reports claiming association between third generation antipsychotics (e.g., aripiprazole) and impulse control disorders were more likely the result of insufficient dosage or undetected comorbidity [35,36]. Hyperdopaminergic and decreased serotonin states have been linked to increase in impulsivity, aggression, and emotional dysregulation. As such, antipsychotics in general have been effective in managing impulsive aggression through dopamine stabilization [37,38]. While patient factors, such as high Hawthorne effect and suicidality may limit studies, the expert panel noted that cariprazine’s mechanism may have a role in borderline personality disorder management, especially in the presence of comorbid depression (bipolar and unipolar), subsyndromal psychosis, and mood instability.

The expert panel considered to continue effective medication after remission of bipolar disorder, if a patient has had multiple relapses. According to the 2021 CANMAT/ISBD Guidelines for managing mixed episodes in bipolar in the maintenance phase, while there are no first- or second-line treatments for these presentations, expert opinion supports the use of third-line treatments following resolution of acute mixed presentation, among which is cariprazine [39].

One limitation of this study is the employment of a structured targeted literature review (TLR) rather than a comprehensive systematic review. In disciplines such as healthcare, public health, and policy development, TLRs are crucial for synthesizing evidence and informing decision-making processes [21]. Similarly, this TLR approach has been utilized by similar consensus development studies in psychiatry [18,19]. Another limitation is that the primary evidence obtained predominantly originates from Western countries. Nevertheless, the retrieved publications were considered adequate to support the creation of clinical scenarios and to inform the panel in assessing the appropriateness of these scenarios. This study did not explore the potential impact of cultural or religious beliefs and public stigma on the management of bipolar disorders. Additionally, patients in Southeast Asian countries primarily finance their healthcare services through direct payments (i.e., out-of-pocket expenses), and affordability was not factored into the formulation of the recommendations. Future research may focus on real-world studies of cariprazine in the Southeast Asia region. Furthermore, subsequent studies might consider investigating the effects of cariprazine in BP1D with psychiatric comorbidities as well as other mood disorders (e.g., bipolar II disorder).

These consensus recommendations are designed to assist psychiatrists and other mental health professionals in managing bipolar disorders with cariprazine. They are meant to complement, rather than replace, clinical judgment and should be applied alongside individual patient needs, preferences, and circumstances. Given the varied healthcare landscapes in Southeast Asia, it is also essential to consider the availability, regulatory status, and affordability of cariprazine in each local context.

## 5. Conclusions

Based on clinical experience and best available evidence, cariprazine is considered a rational option for the treatment of the full spectrum of bipolar I disorder. Cariprazine offers a more favorable metabolic profile and demonstrates comprehensive efficacy in addressing both acute mania and bipolar depression, in comparison to most atypical antipsychotics. The consensus recommendations may serve as practical guidance to assist clinicians in making informed decisions regarding the management of adult patients with BP1D, while taking into account the specific needs, preferences, and circumstances of individual patients.

## Figures and Tables

**Table 1:** Consensus statements on the appropriate clinical utility of cariprazine in the management of bipolar I depression.

	Indications	Rating	Median (IQR)
	For an adult patient diagnosed with bipolar 1 depression,		
1	… cariprazine monotherapy is preferred in most cases	Appropriate	8 (8–9)
2	… cariprazine combination therapy with other mood stabilizers (e.g., lithium, divalproex) is suitable in certain scenarios.	Appropriate	8 (7–9)
3	… cariprazine is suitable as a first-line treatment.	Appropriate	8 (7–9)
4	… cariprazine is suitable in first episode bipolar 1 depression.	Appropriate	8 (7–9)
	For an adult patient diagnosed with bipolar 1 depression, cariprazine is suitable for patients with:		
5	… suicidal ideation or behavior	Appropriate	8 (7–9)
6	… cognitive symptoms (e.g., problems with concentration, mental calculation, solving problems, learning new information)	Appropriate	9 (8–9)
7	… functional impairment	Appropriate	8 (8–9)
8	… partial adherence or non-adherence to previous medications	Appropriate	8 (7–9)
9	… older age (>65 years old)	Appropriate	8 (6–9)
10	… anhedonia	Appropriate	8 (6–9)
	For an adult patient diagnosed with bipolar 1 depression, cariprazine is suitable for patients with the following clinical features:		
11	… need for rapid response is required, e.g., patients at risk of suicide, with psychotic features, or who have medical complications, including dehydration	Appropriate	8 (7–9)
12	… anxious distress	Appropriate	8 (7–9)
13	… mixed features	Appropriate	8 (8–9)
14	… rapid cycling	Appropriate	8 (7–9)
15	… psychotic features	Appropriate	8 (7–9)
16	… melancholia features	Appropriate	8 (7–9)
17	… atypical features	Appropriate	8 (7–9)
18	… postpartum onset (without breastfeeding)	Appropriate	8 (6–9)
19	… catatonia	Appropriate	7 (2–9)
	For an adult patient diagnosed with bipolar 1 depression, cariprazine is suitable for patients with the following co-morbidities:		
20	… substance use disorder	Appropriate	8 (7–9)
21	… impulse control disorders	Appropriate	8 (6–9)
22	… anxiety disorders	Appropriate	8 (7–9)
23	… obsessive compulsive disorder	Appropriate	8 (7–9)
24	…. ADHD	Appropriate	7 (5–9)
25	… personality disorders	Appropriate	8 (6–9)
26	… metabolic disorders	Appropriate	7 (7–9)

**Table 2:** Consensus statements on the appropriate clinical utility of cariprazine in the management of acute mania/mixed episode.

	Indications	Rating	Median (IQR)
	For an adult patient diagnosed with bipolar 1 disorder in an acute mania/mixed episode,		
1	… cariprazine monotherapy is preferred in most cases.	Appropriate	8 (8–9)
2	… cariprazine combination therapy with other mood stabilizers (e.g., lithium, divalproex) is suitable in certain scenarios.	Appropriate	8 (8–9)
3	… cariprazine is suitable as a first-line treatment.	Appropriate	8 (8–9)
4	… cariprazine is suitable in first episode mania.	Appropriate	8 (8–9)
	For an adult patient diagnosed with bipolar 1 disorder in an acute mania/mixed episode, cariprazine is suitable for patients with:		
5	… suicidal ideation or behavior	Appropriate	8 (8–9)
6	… agitation as monotherapy	Appropriate	8 (8–9)
7	… agitation in combination with benzodiazepine	Appropriate	9 (8–9)
8	… cognitive symptoms (e.g., problems with concentration, mental calculation, solving problems, learning new information)	Appropriate	9 (8–9)
9	… functional impairment	Appropriate	8 (8–9)
10	… partial adherence or non-adherence to previous medications	Appropriate	8 (8–9)
11	… older age (>65 years old)	Appropriate	8 (7–9)
12	… anhedonia	Appropriate	8 (7–9)
	For an adult patient diagnosed with bipolar 1 disorder in an acute mania/mixed episode, cariprazine is suitable for patients with the following clinical features:		
13	… need for rapid response is required, e.g., patients at risk of suicide, with psychotic features, or who have medical complications, including dehydration.	Appropriate	8 (8–9)
14	… anxious distress	Appropriate	8 (8–9)
15	… mixed features	Appropriate	8 (8–9)
16	… concurrent depressive symptoms	Appropriate	8 (8–9)
17	… rapid cycling	Appropriate	8 (8–9)
18	… psychotic features	Appropriate	8 (7–9)
19	… hostility	Appropriate	8 (8–9)
20	… irritability/disruptive-aggressive behavior	Appropriate	8 (8–9)
21	… catatonia	Appropriate	8 (8–9)
22	… postpartum onset (without breastfeeding)	Appropriate	8 (7–8)
	For an adult patient diagnosed with bipolar 1 disorder in an acute mania/mixed episode, cariprazine is suitable for patients with the following co-morbidities:		
23	… substance use disorder	Appropriate	8 (7–9)
24	… impulse control disorders	Appropriate	8 (7–9)
25	… anxiety disorders	Appropriate	8 (7–9)
26	… obsessive compulsive disorder	Appropriate	8 (7–9)
27	…. ADHD	Appropriate	8 (7–8)
28	… personality disorders	Appropriate	7 (6–8)
29	… metabolic disorders	Appropriate	8 (7–8)

**Table 3:** Suggestions for lower and higher dose of cariprazine in the treatment of bipolar depression.

**Suggestions for Lower Dose**	**Suggestions for Higher Dose**
In mild to moderate cases, as an initial dose, particularly with treatment-naïve patients and with those who achieve a good or adequate response to low doses Adverse event profile: patient sensitivity, history of akathisia Patient preference	Moderate to severe depression with agitation or psychotic features Poor or inadequate treatment response to lower dose Bipolar depression with concurrent manic symptoms

**Table 4:** Current practices in management of TEAES associated with cariprazine.

**Treatment-Emergent Adverse Events Associated with Cariprazine**	**Current Practices**
Tardive Syndromes	
Tardive syndromes, i.e., akathisia, parkinsonism, dystonic reactions	Dose reduction Switch to alternative antipsychotic Discontinuation of medication Augmentation Specific to akathisia: Wait and see Prevention: slower titration Augmentation with: o Beta-blockers e.g., propranolol 30–80 mg in three divided dose/day o Benzodiazepines o Mirtazapine o Vitamin B6
Sleep Disturbances	
Insomnia	Change the time of medication—give in the daytime Use of melatonin, sedative-hypnotics, dual orexin-2 antagonists, or antihistamines Benzodiazepine as last option for insomnia management
Somnolence/sedation	Change time of medication—give at night Use lower dose Slow titration Switch medication Addition of modafinil to improve daytime alertness

## Data Availability

The original contributions presented in this study are included in the article. Further inquiries can be directed to the corresponding author.

## Abbreviations

The following abbreviations are used in this manuscript:

α1/α2	Alpha 1/Alpha 2
5HT1A	5-hydroxytryptamine (serotonin) receptor 1A
BP-1D	Bipolar 1 Disorder
CANMAT	Canadian Network for Mood and Anxiety Treatments
CPG	Clinical Practice Guidelines
D2/D3	Dopamine D2/Dopamine D3
FAST	Reisberg Functional Assessment Screening Tool
GABA	Gamma-aminobutyric acid, γ-aminobutyric acid
HAM-A	Hamilton Anxiety Rating Scale
IQR	Interquartile range
ISBD	International Society for Bipolar Disorders
MADRS	Montgomery–Åsberg Depression Rating Scale
PANSS	Positive Scale, Negative Scale, and General Psychopathology Scale
RAM	RAND/UCLA Method
RAND/UCLA	RAND Corporation/University of California Los Angeles
RCBD	Rapid cycling bipolar disorders
TEAE	Treatment emergent adverse events
TLR	Targeted literature review
